# A rare case of cutaneous leishmaniasis in Kathmandu presenting with features of bacterial skin infection: A case report

**DOI:** 10.1002/ccr3.7029

**Published:** 2023-03-11

**Authors:** Anil Regmi, Biplov Adhikari, Pragya Karki, Suwash Baral, Irusha Rani Adhikari

**Affiliations:** ^1^ Nepal Medical College Teaching Hospital Kathmandu Nepal; ^2^ Everest Hospital Pvt. Ltd. Kathmandu Nepal; ^3^ Anandaban Leprosy Hospital Lalitpur Nepal

**Keywords:** amastigote, cutaneous leishmaniasis, Nepal, skin infection

## Abstract

Leishmaniasis is an infectious disease caused by different species of genus Leishmania and transmitted by sandflies. Lesions of CL are most commonly present in the exposed areas, and the most familiar morphological type is papulo‐nodular. The diagnosis of CL should be considered while dealing with common skin lesions, as well as encountering uncommon pathologies. We present a case of a 26‐year‐old man living in Kathmandu originally from Humla whose clinical course was complicated by unsuccessful treatment with suspicion of bacterial skin infection. The patient first presented with an erythematous papule with some scale and crust with central ulceration over the left side of his upper lip and mild fever. With the suspicion of bacterial infection, he was initially treated with antibiotics, which showed no improvement prompting the referral to a tertiary center with further diagnostic workup. Punch biopsy confirmed the presence of amastigote form of leishmaniasis Donovan bodies. Also, the rk39 antibody test was positive. Clinicians need to pay more effort to the diagnosis of CL and include it in the differential diagnoses of patients presenting with typical lesions even if the region is not known to be endemic for CL or in the patient with no known history of insect bite.

## Graphical abstract

Clinicians need to pay more effort to the diagnosis of CL and include it in the differential diagnoses of patients presenting with typical lesions even if the region is not known to be endemic for CL or in the patient with no history of insect bites.

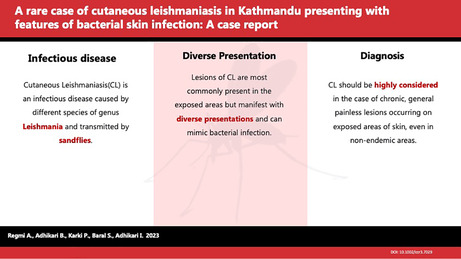

## 1 INTRODUCTION

Leishmaniasis is an infectious disease caused by different species of genus Leishmania and transmitted by sandflies. The protozoans get transmitted by two genera of sandfly vectors, *Phlebotomus* and *Lutzomyia*.^1^ Leishmania can be found in a variety of mammalian reservoirs.^2^ Leishmaniasis comprises a spectrum of diseases with three major clinical subtypes: cutaneous, mucocutaneous, and visceral. Leishmaniasis can also be classified geographically based on the predominant area it occurs. Cutaneous leishmaniasis is the most common form globally. It is the most common form in Nepal too, and the number of reported cases is rising in recent years.^3^ Cutaneous leishmaniasis is endemic in developed countries, including the United States and other European countries.^2^, ^4^

Lesions of CL are most commonly present in the exposed areas, and the most familiar morphological type is papulo‐nodular.^5^ However, CL can manifest with diverse presentations and can be seen in unusual sites or have atypical morphologies.^6^ Thus, the diagnosis of CL should be considered while dealing with common skin lesions, as well as encountering uncommon pathologies.^6^ There is not one generalized treatment regimen for CL as therapy should be individualized based on the patient, presentation, and parasite.^3^, ^4^ We present a case of a 26‐year‐old man living in Kathmandu originally from Humla. He was successfully treated with 1 monthly course of oral Miltefosine.

## 2 CASE PRESENTATION

**FIGURE 1:**
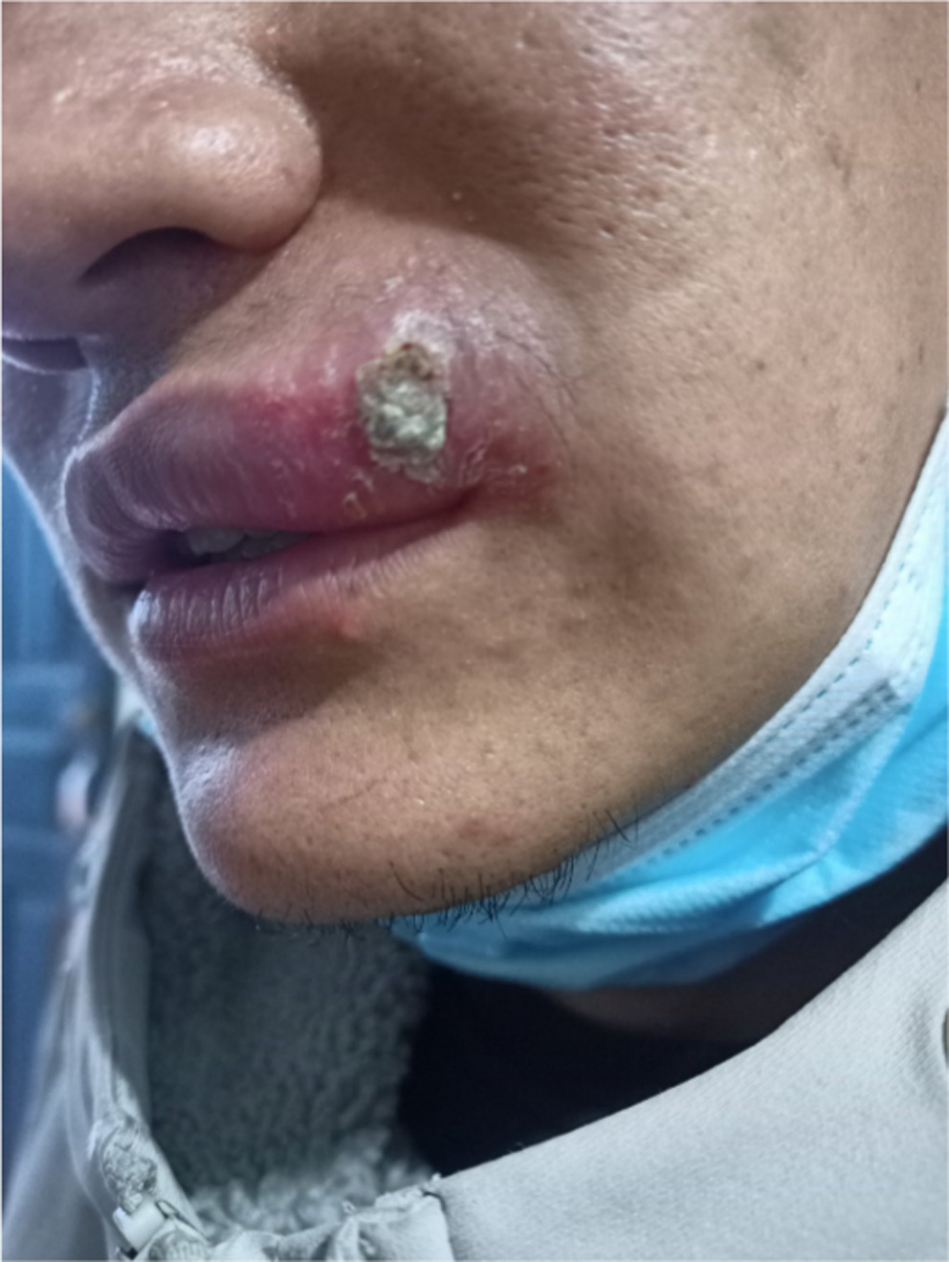
Lesion before the start of treatment.

**FIGURE 2:**
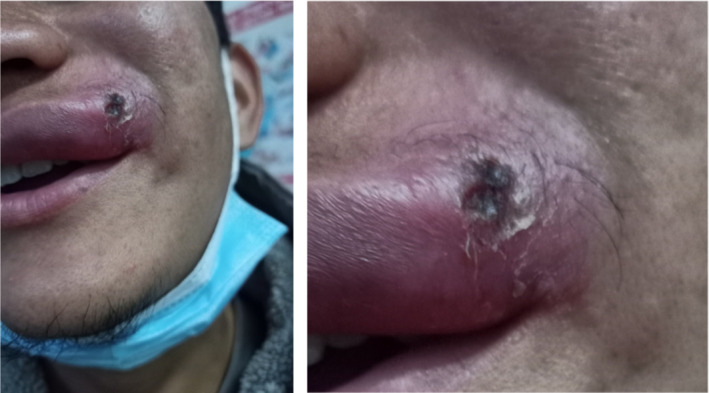
Lesion after 5 days of initiation of treatment.

**FIGURE 3:**
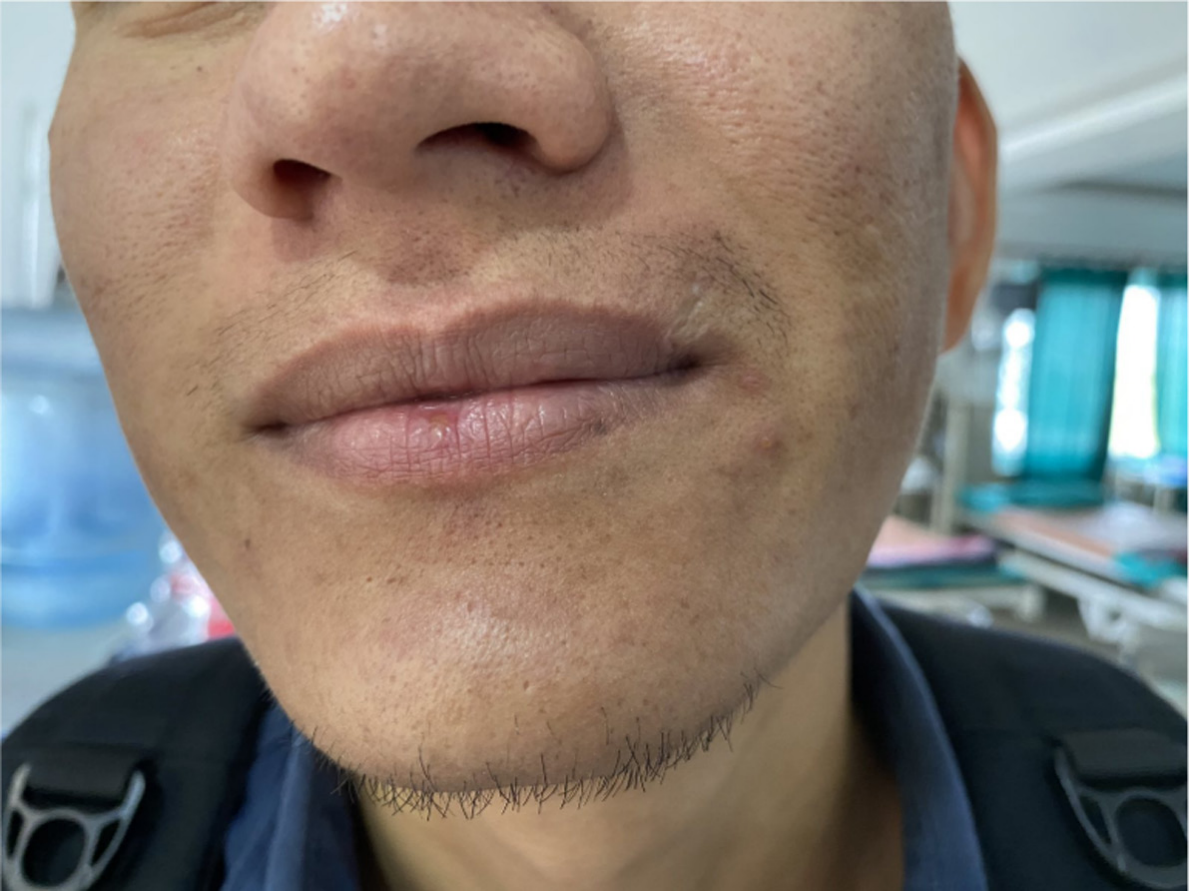
After completion of therapy.

A previously healthy 25‐year‐old man presented to Thankot primary hospital with a history of skin lesions over the left side of his upper lip and mild fever for the past 4 weeks (Figure 1). The patient described the lesion as painless, gradually increasing in size, red, mild, and nonhealing with serous discharge. The patient had a history of frequent episodes of herpes labialis but no other significant past or family history of note. The physical examination revealed left‐sided level I and right‐sided level II lymphadenopathy. Besides the lesion, the complete skin along with the examination of the nares, septum, and oropharynx was unremarkable. An erythematous papule with some scale and crust with central ulceration was found on the examination of the lesion (Figure 2). A course of flucloxacillin antibiotic along with mupirocin ointment and acetaminophen was prescribed with a possible diagnosis of bacterial infection of the skin, which did not resolve his symptoms. The patient had been residing in Humla, Karnali, an endemic region of CL before he migrated to Kathmandu 1 year ago. However, he did not recall any insect bite or visit any other region during the past 1 year. The patient was then referred to a tertiary hospital where a slit smear examination of the lesion was done for CL and acid‐fast Bacilli. However, both of the results were negative. Furthermore, FNAC and eventually punch biopsy were done, which confirmed the presence of amastigote form of leishmaniasis Donovan bodies. The rk39 antibody test was also performed, which was positive, but the PCR, a highly sensitive and specific test for CL, appeared negative. The patient was prescribed oral Miltefosine 50 mg three times daily for 4 weeks after which his lesion was healed with an atrophic scar (Figure 3).

## 3 DISCUSSION

According to Pandey et al., 42 cases of CL were diagnosed in 26 different hospitals in Nepal between 2016 and 2019 based on clinical presentations and laboratory findings (demonstration of amastigotes in Giemsa‐stained smears and rK39 test results).^7^ The common features of the patients in the study and this case are the predominance of the lesion in the facial region (38.1%, 16/42) and successful treatment with Miltefosine for 28 days. Although Nepal is considered nonendemic for CL where most of the programs are focused on eliminating visceral leishmaniasis, it is highly imperative to consider the emergence of CL in Nepal. Choice of the test to accurately diagnose CL should be considered based on the sensitivity and specificity of a test and more importantly, on the availability.^8^ This is especially more significant in a country like Nepal, where resources are limited. Early diagnosis and prompt treatment of patients with CL have been difficult due to the rare nature of the disease and nonspecific presentation.^8^ The differential diagnosis of CL should be highly considered in the case of chronic, general painless lesions occurring on exposed areas of skin, especially in people living in endemic areas.^9^

## 4 CONCLUSION

Clinicians need to pay more effort on diagnosing the cases of CL and include it in the differential diagnoses of patients presenting with typical lesions even if the region is not known to be endemic for CL or in the patient with no known history of insect bite. This may increase the rate of timely diagnosis of cases of CL in Nepal with prompt treatment reducing the patient suffering and diagnostic dilemma. This case report has been written in line with the SCARE 2020 criteria.^10^

## AUTHOR CONTRIBUTIONS

**Anil Regmi:** Conceptualization; data curation; formal analysis; writing – original draft; writing – review and editing. **Biplov Adhikari:** Conceptualization; data curation; formal analysis; writing – original draft. **Pragya Karki:** Formal analysis; supervision; writing – original draft. **Suwash Baral:** Conceptualization; investigation; writing – original draft. **Irusha Rani Adhikari:** Conceptualization; resources; writing – original draft.

## FUNDING INFORMATION

N/A

## CONFLICT OF INTEREST STATEMENT

None.

## ETHICAL APPROVAL

According to the local ethical guideline, ethical approval is not necessary to write a case report. We obtained written consent from the patient to include the clinical details including pictures.

## CONSENT

Written informed consent was obtained from the patient for the publication of this case report and accompanying images. A copy of the written consent form is available for review by the Editor‐in‐Chief of this journal upon request.

## PROVENANCE AND PEER REVIEW

Not commissioned, externally peer‐reviewed.

## Data Availability

Data sharing is not applicable to this article as no new data were created or analyzed in this study.
